# Phenotypic Variability and Genetic Diversity Analysis of Chickpea (*Cicer arietinum* L.) Germplasm Resources

**DOI:** 10.3390/plants15010024

**Published:** 2025-12-21

**Authors:** Shuping Zhang, Jundong Su, Wanming Li, Lili Xue, Xuefei Cai, Tingzhao Li, Jing Xiao, Jinbo Zhang

**Affiliations:** 1 College of Life Science and Technology, Xinjiang University, Urumqi 830046, China; zhangsp0103@163.com (S.Z.); liwanming666@126.com (W.L.); 2 State Key Laboratory of Ecological Safety and Sustainable Development in Arid Lands, Xinjiang Institute of Ecology and Geography, Chinese Academy of Sciences, Urumqi 830011, China; sunasu1112@163.com (J.S.); x1465452227@outlook.com (L.X.); 3 China-Tajikistan Belt and Road Joint Laboratory on Biodiversity Conservation and Sustainable Use, Xinjiang Institute of Ecology and Geography, Chinese Academy of Sciences, Urumqi 830011, China; 4 Amway (China) Botanical R&D Center, Wuxi 214111, China; carl.cai@amway.com (X.C.); teric.li@amway.com (T.L.); 5 Institute of Crop Sciences, Xinjiang Academy of Agricultural Sciences, Urumqi 830091, China

**Keywords:** chickpea, germplasm resources, agronomic traits, quality traits, genetic diversity

## Abstract

This study evaluated 362 chickpea accessions by analyzing the phenotypic variation of 17 major traits. The main agronomic traits and quality traits were comprehensively evaluated using principal component analysis (PCA) and cluster analysis. The results revealed a Shannon diversity index (*H*’) for the five qualitative traits ranging from 0.76 to 1.20, while for the twelve quantitative traits, it ranged from 1.45 to 2.07. The coefficient of variation (CV) ranged from 7.63% to 41.69%, demonstrating substantial variation and significant differences among the 362 germplasm resources. Correlation analysis revealed that traits such as growth period, plant height, seed weight per plant, and hundred-seed weight were closely correlated with yield. PCA extracted five principal components, collectively explaining 76.06% of the total variance, representing most of the agronomic traits and quality traits. Cluster analysis categorized the accessions into five distinct groups, which can be used as germplasm alternative materials with high yield, mechanization potential, large grain size, early maturity, stress resistance, and high protein content. Using a membership function, a comprehensive evaluation score (F-value) was calculated, leading to the identification of ten accessions with superior overall traits. These could be used as materials for breeding and germplasm creation of new chickpea varieties. This research provides a scientific basis for future parental selection in chickpea breeding programs and for the screening of specific chickpea germplasm resources.

## 1. Introduction

Chickpeas (*Cicer arietinum* L.) are an annual plant belonging to the legume family (Fabaceae) and the genus *Cicer*. According to market classifications, commercial chickpeas are primarily categorized into two seed types: desi and kabuli [1]. As the third-largest legume crop globally, chickpeas are cultivated and consumed in over 50 countries. Desi chickpeas predominate in countries such as India and Iran, accounting for approximately 80–85% of the global cultivation area. In contrast, the kabuli type is primarily cultivated in South Asia, East Africa, and Australia [2]. In China, chickpeas are mainly grown in Xinjiang, Gansu, Qinghai, and Yunnan provinces, with Xinjiang alone contributing to over 85% of the national planting area [3]. Chickpeas are highly nutritious, rich in protein, carbohydrates, fats, both soluble and insoluble fibers, vitamins, and folic acid, while being cholesterol-free [4,5]. Furthermore, as a nitrogen-fixing crop, chickpeas can reduce emissions of the greenhouse gas nitrous oxide and enhance soil fertility, thus holding significant ecological and economic value in agricultural production systems [6].

Germplasm resources form the material foundation for innovation in the seed industry and are strategic assets for ensuring national food security [7]. Globally, significant efforts have been made to conserve and characterize the genetic diversity of chickpeas. For example, the ICRISAT gene bank holds 20,838 chickpea accessions from 59 countries, representing approximately 20.8% of global collections, and has established both a core collection of 1954 and a mini-core collection of 211 accessions. Genomic studies have further confirmed the rich genetic diversity present in these resources [8,9]. However, chickpeas possess a relatively narrow genetic background, and long-term domestication has constrained its resistance to biotic and abiotic stresses, such as diseases and drought, as well as its yield potential, thereby hindering breeding progress [10]. Therefore, an in-depth investigation into the phenotypic variation and genetic diversity of key agronomic traits in the chickpea germplasm is crucial for breeding high-yield, high-quality, and stress-resistant varieties. Several studies have systematically evaluated the phenotypic and genetic diversity of the chickpea germplasm. Yu et al. [11] analyzed 12 agronomic traits across 181 accessions and reported coefficient of variation (CV) ranging from 7.47% to 85.26%. Traits such as hundred-seed weight, number of branches, number of pods, flowering time, yield, yield per plant, and number of unfilled pods all exhibited CVs exceeding 30%, indicating strong potential for improvement. Similarly, Shao et al. [12] assessed five phenotypic traits in 151 accessions and found that 100-seed weight had the highest diversity index (7.750), followed by pods per plant (7.387). In a study of 103 germplasm accessions, Huang et al. [13] performed genetic diversity and cluster analyses based on 12 agronomic traits and classified the materials into five distinct groups. Group I (24 accessions) was identified as a source of high-yielding lines suitable for mechanized harvesting, while Group II (13 accessions) contained high-yielding, tall-stemmed, medium-grain types. Principal component analysis revealed that the first five components collectively accounted for 79.99% of the total variation. Liu et al. [14] evaluated 114 germplasm lines for 15 agronomic traits and identified five elite accessions—‘86-68c’, ‘2014-088’, ‘Zhang hei dao dou’, ‘Zhang gao chan nao dou’, and ‘87pin-33’—with desirable characteristics such as optimal plant height, high yield, diverse seed color, and large seed size. In another evaluation, Hao et al. [15] assessed 13 traits in 160 chickpea introductions from ICRISAT and selected 63 specific germplasm accessions with early maturity, dwarfism, multi-podding, large seeds, and high yield potential. Collectively, these studies provide a valuable foundation for chickpea breeding by identifying key traits and superior germplasm. Building on this foundation, this study aimed to fully harness the potential of existing chickpea germplasm resources. To this end, 362 chickpea accessions from various countries and regions were utilized as research materials to systematically analyze genetic diversity and the correlation of phenotypic traits. Principal component analysis and cluster analysis were employed to evaluate agronomic and quality traits, with the goal of identifying superior germplasm resources. This research provides a theoretical basis for the efficient utilization of high-quality chickpea germplasm for guiding breeding strategies, thereby broadening the crop’s genetic base. Furthermore, this work proposes new directions for quality breeding in chickpea, which is expected to accelerate the breeding process.

## 2. Results

### 2.1. Genetic Diversity Analysis of Main Agronomic Traits

An analysis of five qualitative and twelve quantitative traits across 362 chickpea germplasm accessions uncovered significant genetic variation. The Shannon-Wiener diversity index (*H*’) for the qualitative traits ranged from 0.758 to 1.203 (Table 1). Seed coat color (SCC) displayed the highest diversity (*H*’ = 1.203), with beige being the most common phenotype (at 66.2%), followed by light brown, brown, reddish-brown, and smaller proportions of black, yellow, and ivory white. Plant type (PT) also demonstrated considerable diversity (*H*’ = 1.123), with the semi-spreading type predominating at 57.4%, followed by erect and semi-erect types. Flower color (FC) was less varied (*H*’ = 0.760), with white flowers being the most prevalent at 76.2%, followed by pink and purplish-red. Seed shape (SS) had the lowest diversity index (*H*’ = 0.758), with the eagle-head shape being the most frequent at 72.8%, followed by the sheep-head and spherical shapes. Lastly, the diversity index for seed surface shape (SSS) was 0.800, where a rough texture was the most common at 67.4%, followed by uneven (25.7%) and smooth (6.9%) textures.

**Figure 1:**
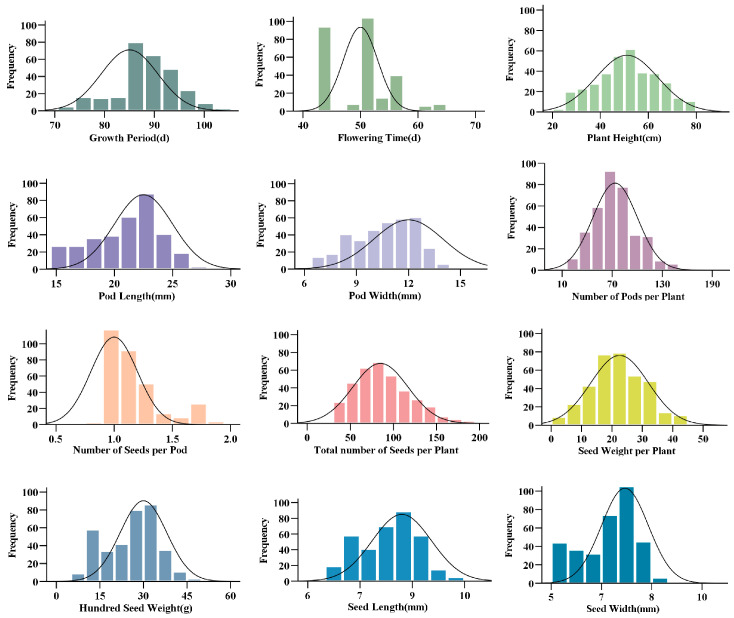
Normal frequency distribution plots of 12 quantitative traits.

Normality assessment of the 12 quantitative traits, conducted via the Kolmogorov–Smirnov’s (K-S) test and visualized by frequency distribution histograms (Figure 1), revealed that plant height (PH) conformed to a normal distribution (*p* > 0.05). In contrast, the remaining traits showed significant departures from normality *(p* < 0.05), although their distributions were apparently normal. Overall, the chickpea germplasm exhibited continuous genetic variation and a relatively even distribution of polymorphisms.

The analysis of 12 quantitative traits across 362 chickpea germplasm accessions uncovered significant genetic variation (Table 2). The coefficient of variation for these traits varied from 7.63% to 41.69%. Notably, the CV for seed weight per plant (SWP) was the highest, ranging from 2.20 to 63.53 g, followed by total number of seeds per plant (TNS), Number of Pods per Plant (NPP), and hundred-seed weight (HSW). The variation coefficients for Pod Length (PL) and Seed Length (SL) were relatively small, both at 10.88%. The variation coefficient for growth period (GP) was the smallest. The Shannon–Weaver diversity index (*H*’) for the 12 quantitative traits of the tested materials ranged from 1.45 to 2.07. PH exhibited the highest diversity, followed by PL and Number of Seeds per Pod (NSP), while GP had the lowest. The broad-sense heritability (*H*^2^) calculated based on four environments ranged from 53.45% to 92.40% across traits, with hundred-grain weight showing the highest heritability.

This study conducted experiments in Urumqi and Baicheng, Xinjiang, China, in 2023 and 2024, respectively. The results of multivariate analysis of variance are shown in Table 3. The years and locations had extremely significant main effects on GP, FT, PH, and PW. The years correspond to PL, and NPP, NSP, SWP, HSW, and SL all have extremely significant main effects and have relatively significant main effects on TNS and SW. The locations are related to NPP; NSP has a relatively significant main effect, but has no significant effect on PL, TNS, SWP, HSW, SL, and SW.

The comprehensive analysis indicates that GP exhibits the lowest coefficient of variation and genetic diversity index, suggesting that this trait has become relatively stable due to long-term directional selection. In contrast, traits associated with yield or quality, such as PL, maintain substantial variation, indicating a greater potential for breeding improvement.

### 2.2. Correlation Analysis of 12 Quantitative Traits

**Figure 2:**
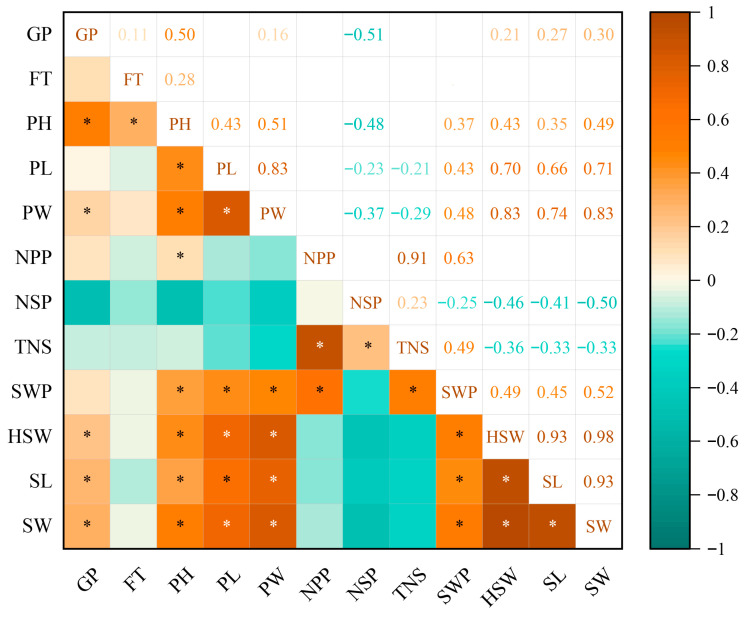
Correlation analysis of major agronomic traits in 362 chickpea accessions. * *p* ≤ 0.05.

We analyzed the correlations among six main agronomic traits in 362 chickpea accessions using Pearson’s method, with significance determined by Student’s *t*-test (Figure 2). Of the various combinations, 31 showed a highly significant positive correlation, constituting 46.97% of all possible combinations. Furthermore, 16 groups exhibited an extremely significant negative correlation, which accounted for 24.24% of the total combinations. One group of combinations had a significant positive correlation, and three groups had a significant negative correlation. The GP trait was significantly positively correlated with Pod Width (PW), PH, HSW, SL, and Seed Width (SW). PH was significantly positively correlated with flowering time (FT), GP, PL, PW, SWP, HSW, SL, and SW. PW was significantly positively correlated with GP, PH, PL, SWP, HSW, SL, and SW. The NPP trait was significantly positively correlated with TNS and SWP. SWP was significantly positively correlated with PH, PL, PW, NPP, TNS, HSW, SL, and SW. Additionally, there was a significant positive correlation between NPP and PH. PL was significantly negatively correlated with TNS. PW was significantly negatively correlated with NPP, NSP, and TNS. The NPP was significantly negatively correlated with HSW and SL. The NSP was significantly negatively correlated with GP, PH, PL, PW, SWP, HSW, SL, and SW. FT was significantly negatively correlated with NSP and SL. The NPP was significantly negatively correlated with PL and SW. Based on the correlation results between each index, PH, NSP, and SWP had the highest frequency of occurrence, and these three traits were also closely related to yield. The number of combinations with significant correlations reached 51 groups, indicating that the quantitative traits were interrelated and mutually constrained.

### 2.3. Principal Component Analysis of Main Agronomic Traits and Quality Traits

Principal component analysis (PCA) of 12 quantitative and 4 quality traits across 362 chickpea germplasm accessions yielded five principal components, accounting for 76.06% of the cumulative variance (Table 4). The first component, accounting for 32.358% of the variance, was defined by a significant negative correlation between PH and NPP, as well as an inverse relationship between grain size and yield components. This highlights a potential trade-off between vegetative growth and reproductive output, suggesting that varieties with larger seeds may produce fewer pods or lower overall yield per plant. The second component contributed 15.908% of the variance. The characteristic values of PL and PW were the largest, and the characteristic values of SL, SW, and SWP were larger, indicating that principal component 2 was related to the coordinated development of pods and seeds. If the morphology of pods and seeds can be optimized, the yield can be increased simultaneously. The contribution rate of third component was 11.240%. The characteristic values of FT, PL, and PW were larger, and the characteristic value of SWP was the smallest, indicating that principal component 3 was mainly related to flowering time and pod development. The contribution rate of the fourth component was 9.993%, and linked pod circumference and a composite of SSCC to yield per plant, suggesting a potential relationship between certain quality traits and yield. Finally, the fifth component (6.565% contribution) was dominated by nutritional quality, where FC and AC were negatively correlated with each other. Collectively, these components dissect the complex trait architecture of chickpeas, providing a quantitative framework for multi-trait selection in breeding programs.

The 12 quantitative traits and 4 quality traits were defined as follows: X_1_, X_2_, X_3_, X_4_, X_5_, X_6_, X_7_, X_8_, X_9_, X_10_, X_11_, X_12_, X_13_, X_14_, X_15_, and X_16_. Using the eigenvalues of each index and their corresponding eigenvectors, the linear combination between these 5 principal components and the 16 indicators is derived as follows:

F_1_ = 0.12X_1_ + 0.046X_2_ − 0.137X_3_ − 0.032X_4_ + 0.014X_5_ − 0.144X_6_ − 0.145X_7_ − 0.173X_8_ − 0.013X_9_ + 0.169X_10_ + 0.153X_11_ + 0.173X_12_ + 0.023X_13_ − 0.013X_14_ − 0.021X_15_ + 0.042X_16_ (1)

F_2_ = −0.178X_1_ − 0.175X_2_ − 0.162X_3_ + 0.282X_4_ + 0.289X_5_ + 0.041X_6_ + 0.005X_7_ + 0.039X_8_ + 0.201X_9_ + 0.131X_10_ + 0.175X_11_ + 0.135X_12_ − 0.148X_13_ − 0.023X_14_ + 0.02X_15_ + 0.095X_16_ (2)

F_3_ = 0.115X_1_ + 0.359X_2_ + 0.127X_3_ + 0.338X_4_ + 0.332X_5_ − 0.142X_6_ + 0.119X_7_ − 0.039X_8_ − 0.214X_9_ − 0.135X_10_ − 0.108X_11_ − 0.112X_12_ + 0.018X_13_ − 0.154X_14_ + 0.106X_15_ + 0.116X_16_ (3)

F_4_ = 0.153X_1_ + 0.063X_2_ + 0.156X_3_ + 0.049X_4_ + 0.054X_5_ + 0.294X_6_ − 0.193X_7_ + 0.191X_8_ + 0.284X_9_ − 0.024X_10_ − 0.057X_11_ − 0.026X_12_ + 0.388X_13_ + 0.077X_14_ − 0.131X_15_ + 0.387X_16_ (4)

F_5_ = −0.02X_1_ − 0.076X_2_ − 0.01X_3_ + 0.153X_4_ + 0.149X_5_ − 0.121X_6_ + 0.162X_7_ − 0.022X_8_ − 0.287X_9_ + 0.035X_10_ + 0.004X_11_ − 0.011X_12_ + 0.101X_13_ + 0.668X_14_
− 0.562X_15_ − 0.044X_16_ (5)

Based on the variance contribution rate of these five principal components, a principal component comprehensive score model is constructed:

F = 0.425F_1_ + 0.209F_2_ + 0.148F_3_ + 0.131F_4_ + 0.086F_5_ (6)

### 2.4. Cluster Analysis of 362 Chickpea Germplasm Resources

**Figure 3:**
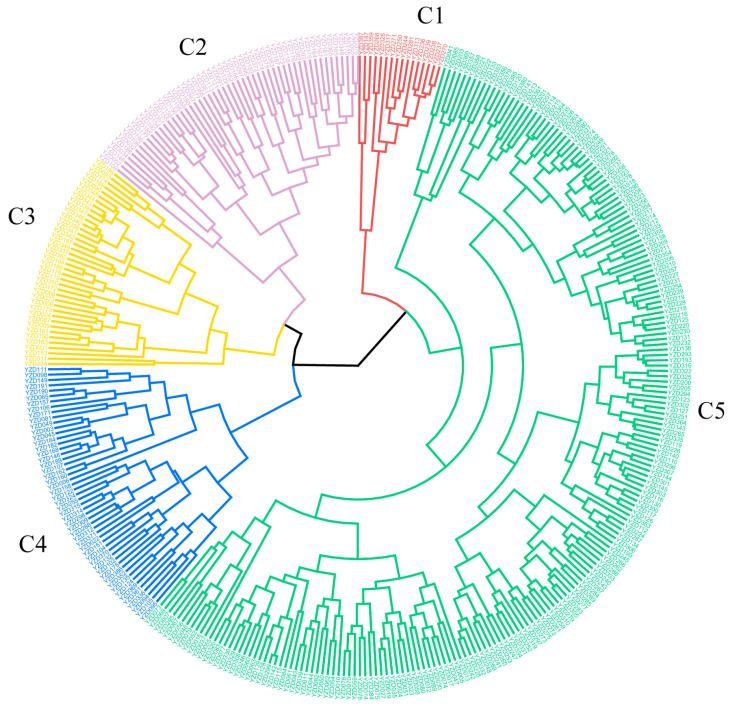
Cluster analysis of 362 chickpea germplasm resources based on 12 quantitative traits and 4 quality traits.

**Figure 4:**
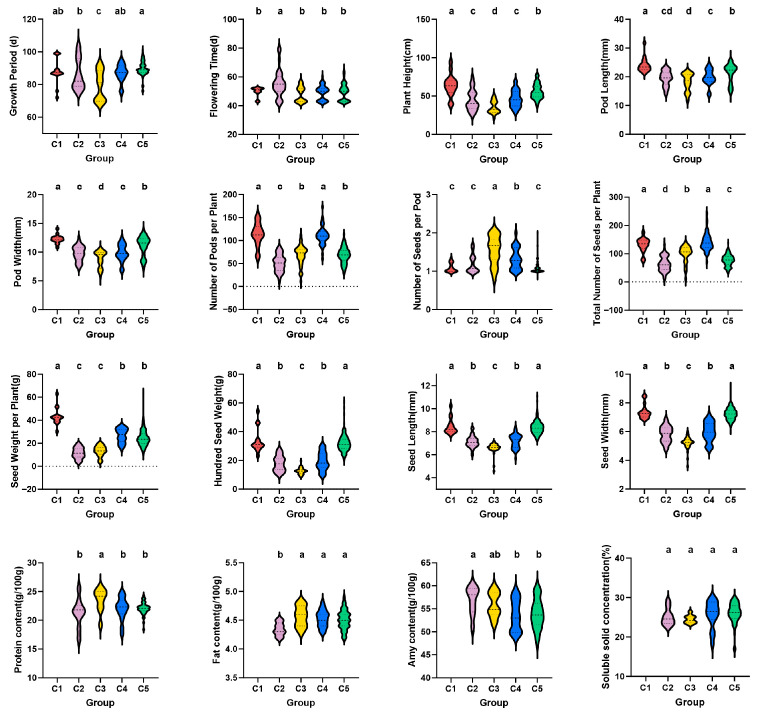
Characteristics of quantitative and quality traits in 5 chickpea groups. Different lowercase letters in the same trait represent significant differences between groups (*p* ≤ 0.05).

Based on standardized data for 12 quantitative and 4 quality traits, hierarchical clustering using the Ward method and Euclidean distance partitioned the 362 chickpea accessions into five groups at a distance threshold of 15 (Figure 3 and Figure 4).

Group C1, comprising 16 kabuli-type chickpea accessions, accounting for 4.42%, was characterized by a relatively high PH, ranging from 58.03 to 94.27 cm. This group also exhibited high values for NPP and TNS, with pods and seeds being large. The NPP was about 110, and the TNS was up to 175. The maximum HSW reached 54.21 g, and NSP ranged from 1 to 2. Notably, six accessions with an erect plant type showed particularly high NPP, TNS, and HSW. Overall, this group had higher PH, more pods per plant, larger grains, and good grain appearance quality.

Group C2, comprising 52 accessions, accounted for 14.36%. This group had a long GP, and the longest GP could reach 105 days. The NPP and the TNS were small, with the NPP being about 50 and the TNS only 11. The SWP was smaller, ranging from 2.20 to 20.10 g. The NPP, TNS, and SWP of this group were small, and the related factors of yield composition were not favorable. Despite these low-yield traits, its unique genetic background presents an opportunity to broaden the genetic base of existing breeding programs.

Group C3, comprising 39 accessions, accounted for 10.77%, mainly consisting of desi type chickpeas. The GP of this group was short, averaging about 79 days. The PH was generally short, ranging from 21.31 to 52.08 cm. The pods and seeds were relatively small, with the HSW ranging from 8.81 to 19.62 g. The PC was higher, and the FC was lower, ranging from 17.00 to 23.60 g/100 g, and about 3.82 g/100 g, respectively. It included 6 materials with semi-erect plant type, short plant height, double flowers, double pods, early maturity, and heat resistance, which could provide materials for the creation of a new germplasm with stress resistance. As an ideal material for breeding early-maturing and heat-resistant varieties, it holds significant potential. This group, comprising resources with high nutritional quality, can leverage its dwarf gene to enhance planting density.

Group C4, comprising 51 accessions, accounted for 14.09%. This group exhibited a remarkably high TNS, reaching a maximum of 243, and a substantial NPP, averaging approximately 110. Although other traits were moderate and the PT was predominantly semi-dispersed, the combination of high pod and seed numbers underscores its unique genetic value. Notably, seven accessions consistently produced over 170 seeds per plant, representing a valuable germplasm for enhancing individual seed-setting ability.

Group C5, the largest group, comprised 204 accessions (56.35%), predominantly of the kabuli type. It was characterized by a low PC, ranging from 15.30 to 22.65 g/100 g, and a high FC of approximately 4.38 g/100 g, and a predominantly semi-draping plant type, followed by erect and semi-erect types. While other traits were moderate, its status as the predominant component of the population makes it a valuable resource for studying environmental adaptability and the evolution of this trait in chickpeas.

### 2.5. Comprehensive Evaluation of Chickpea Germplasm Resources and Screening of Excellent Resources

According to the principal component comprehensive score model, 362 chickpea accessions were ranked, with scores ranging from 0.012 to 0.483, the score information of all chickpea accessions were listed in Table S1. Among them, the top 10 accessions with comprehensive scores were TaYin YZD-2012-22, MuLei (India), 21412(B), Nepal-2, PinYing 1, MuYing 1, Y Zi 14-124-132, MuLei Dali, 21302, and L657 (Table 5).

The top 10 varieties included 9 kabuli chickpeas and 1 desi chickpea, which were excellent chickpea germplasm resources with more grains per plant, larger grains, more prominent yield traits, and higher starch content and soluble solids content. Among them, MuYing 1 is the main cultivar produced in the MuLei area in Xinjiang in the past 10 years. PinYing 1 was the core variety selected by our team. The hybrid combination with PinYing 1 as the parent showed significant heterosis, which provides important genetic resources for the creation of new varieties. 21412 (B) is a desi chickpea with a reddish-brown seed coat and upright plant type. Unlike most other desi chickpeas, 21412 (B) has a longer growth period and more prominent grain traits. The germplasm, with its excellent comprehensive score, showed significant advantages in yield and nutritional quality, which could provide scientific basis and guidance for future chickpea breeding work such as creating a new germplasm and establishing breeding systems.

## 3. Discussion

Genetic diversity within chickpea germplasm is fundamental to breeding progress. Harnessing this potential relies on the comprehensive and accurate evaluation of high-yielding accessions. Such evaluations serve a dual purpose: they provide diverse parental materials for breeding programs and enable the elucidation of the genetic basis underlying complex agronomic traits. Ultimately, this work bridges the gap between applied variety development and fundamental scientific inquiry into chickpea biology.

### 3.1. Genetic Diversity Analysis of Chickpea Germplasm Resources

Phenotypic variation and genetic diversity are foundational metrics for assessing the genetic potential of germplasm, as they reflect trait differentiation and provide insights into a population’s genetic structure. Generally, greater levels of this variation typically signify a richer genetic basis and enhanced potential for selection [16]. In this study, we quantified phenotypic variation across 12 quantitative traits in 362 chickpea accessions using the CV. The CVs ranged widely from 7.63% for GP to 41.69% for SWP. Notably, six key yield traits—PH, NPP, NSP, TNS, SWP, and HSW—all exhibited CVs exceeding 20.00%. This substantial variation, particularly in yield components, demonstrates the rich phenotypic diversity within this collection and underscores its considerable potential for genetic improvement.

Genetic diversity within a germplasm is the cornerstone of crop breeding research [17], and a higher genetic diversity index, such as the Shannon–Wiener index (*H*’), indicates abundant allelic variation and polymorphism [18,19]. In this study, the *H*’ for the 12 quantitative traits ranged from 1.45 (for GP) to 2.07 (for PH), suggesting distinct genetic architectures, while for five quality traits, it varied from 0.76 to 1.20. Both trait categories exhibited substantial genetic variation, confirming abundant allelic diversity in this germplasm collection.

Significant year-to-year variations were observed for the majority of chickpea traits. This effect is likely attributable to the distinct climatic conditions between the two years, with variations sufficient to impact the entire plant lifecycle—from germination and vegetative growth to flowering and seed set. In contrast, the influence of location was primarily mediated by edaphic factors, such as soil fertility, physical structure, and microbial communities, which collectively determine plant vigor and reproductive capacity. Conversely, traits such as PL, TNS, SWP, HSW, SL, and SW appeared to be more dependent on the plant’s inherent genetic potential or were more susceptible to annual environmental fluctuations (light and temperature during the grain-filling period) than to the specific location.

Qualitative traits exhibited lower coefficients of variation and diversity indices than quantitative traits, indicating stronger artificial selection. The phenotypic diversity, correlating with the accession diverse geographical origins, underscores the value of this germplasm collection. To utilize this potential, future breeding should enhance yield by selecting key components and generate novel variation by broadening the genetic base.

### 3.2. Relationships and Cluster Analysis of Quantitative and Quality Traits in Chickpea Germplasm

Quantitative traits in chickpea are polygenic. Phenotypic correlation analysis revealed extensive significant correlations among most traits. Specifically, HSW was positively correlated with PL, PW, SL, SW, and SWP, indicating pod morphology is closely associated with SW. Furthermore, TNS, NPP, PH, and SWP were positively correlated, implying these traits contribute to higher yield [20]. Collectively, these primary yield determinants warrant simultaneous selection in breeding programs.

Cluster analysis is a fundamental method for elucidating genetic relationships and origins within crop germplasm, as it effectively classifies individuals and reveals their correlations [21,22]. This approach has been extensively applied in evaluating germplasm resources of major crops, including soybean (*Glycine max*) [23], rice (*Oryza sativa*) [24], and wheat (*Triticum aestivum*) [25], serving as an effective tool for screening elite breeding populations. In this study, 362 chickpea germplasm accessions were systematically clustered into five distinct groups based on 12 quantitative and 4 qualitative traits. Group C1 was characterized by superior yield-related traits, including high NPP, TNS, HSW, and PH. Notably, six kabuli type chickpea accessions, L633, L634, L635, L636, L645, and L666, exhibited an erect growth habit combined with high values for these yield components, representing a promising germplasm for developing high-yielding varieties suitable for mechanized harvesting. In contrast, Group C2 was characterized by undesirable agronomic traits, namely a prolonged GP, and low NPP and TNS. Conversely, Group C3 exhibited a short GP and favorable quality traits. Six desi type chickpea accessions, 96-010, 96-027, 96-033, 96-044, 96-059, and 96-111, were identified as semi-erect, short in stature, double-flowered, double-podded, early-maturing, and heat-tolerant, making them ideal parental candidate materials for stress-resistance breeding and novel germplasm development.

Group C4 was notable for its high TNS. Seven accessions in particular—three kabuli types (HaYin 2013-ZYD-2, Yin K158 (B), and Yin K157 B) and four desi types (Yin K142, ICCV89323B, L1294, and ICCV89334)—each produced over 170 seeds per plant, highlighting their value as germplasm for enhancing single-plant yield. As the largest subgroup, Group C5 constitutes a valuable resource for investigating the genetic background, adaptive evolution, and core germplasm construction of chickpeas. Overall, cluster analysis provides an intuitive understanding of trait similarities among different accessions, thereby enabling more precise breeding objectives, improving efficiency, and providing a scientific basis for the targeted utilization of specific resources.

### 3.3. Principal Component Analysis and Comprehensive Evaluation of Phenotypic and Quality Traits in Chickpea Germplasm

Principal component analysis (PCA) was employed to reduce dimensionality [26,27]. Applied to 12 quantitative and 4 qualitative traits, PCA extracted five PCs accounting for 76.064% of total variance. A multi-criteria decision-making model was then constructed by integrating PCA with the membership function method [28]. This model standardized raw data and calculated a comprehensive evaluation score (F-value), where a higher value indicates superior performance. Based on this model, 10 accessions with the highest F-values were identified as elite germplasm: TaYin YZD-2012-22, MuLei (India), 21412 (B), Nepal-2, PinYing 1, MuYing 1, Y Zi 14-124-132, MuLeiDali, 21302, and L657. Among these, nine were kabuli types and one was a desi type (L657). The selected accessions exhibited superior agronomic traits, such as high grain number per plant, large seed size, and outstanding yield potential, along with excellent quality characteristics, including high starch and soluble solids content.

The Xinjiang region of China, with its temperate continental climate characterized by abundant sunshine and a significant diurnal temperature range, offers an ideal environment for chickpea cultivation. Expanding the cultivation of this crop can diversify local agricultural systems, create new income streams for farmers, and stimulate the development of ancillary industries. Furthermore, leveraging modern biotechnologies will enable the breeding of novel chickpea varieties specifically adapted to the Xinjiang environment, boasting higher yields and superior quality.

## 4. Materials and Methods

### 4.1. Plant Materials

**Figure 5:**
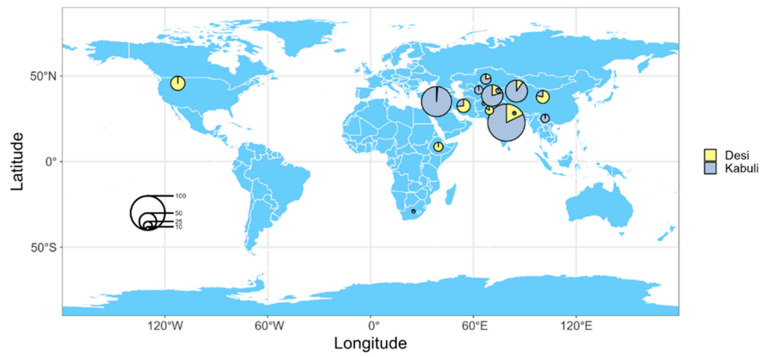
The origin of the 362 chickpea germplasm accessions.

The 362 chickpea germplasm accessions were mainly provided by the National Central Asian Characteristic Crop Germplasm Resources Medium-term Gene Bank (Urumqi), Table S1 provides the source information and seed types of the chickpea materials used in this study. Among the tested chickpea materials, 273 were kabuli types and 89 were desi types, mainly from 18 countries and regions (Figure 5), including 118 from India, 77 from Syria, 15 from Iran, 39 from Tajikistan, 61 from China, 6 from Uzbekistan, 17 from the United States, 1 from Turkey, 1 from Afghanistan, 7 from Ethiopia, 6 from Pakistan, 9 from Kazakhstan, 2 from Kyrgyzstan, 1 from South Africa, 1 from Nepal, and 1 from the former Soviet Union.

### 4.2. Design of Experiment

The experiment design at two locations of the Xinjiang Academy of Agricultural Sciences during the 2023 and 2024 growing seasons: the Anningqu Experimental Station (43°57′ N, 87°29′ E, Urumqi, Xinjiang, China) and the Baicheng Comprehensive Experimental Station (41°47′ N, 81°54′ E, Baicheng, Xinjiang, China). Both sites were characterized by high soil fertility, and standard agronomic practices were implemented throughout the trial period. The preceding crops were wheat and corn. According to the local temperature, given that the lowest temperature was not less than 10 °C, seeds could be sown; sowing was performed annually between late April and early May. The experiment adopted a randomized block design, and two biological replicates were set. Each material was planted in a plot, and three rows were planted in each plot. The row length was 2 m, the row spacing was 40 cm, the plant spacing was 15 cm, the plot spacing was 50 cm, and protective rows were set around.

### 4.3. Investigation of Main Agronomic Traits

Following the Chickpea Germplasm Resources Description Specification and Data Standard [29], 19 main agronomic traits of 362 chickpea germplasm were investigated, including 5 qualitative traits and 12 quantitative traits (Table 6). The qualitative traits included plant type, flower color, seed shape, seed coat color and seed surface shape. The quantitative traits mainly included growth period, flowering time, plant height, Pod Length, Pod Width, Number of Pods per Plant, Number of Seeds per Pod, total number of seeds per plant, seed weight per plant, hundred-seed weight, Seed Length, and Seed Width. The GP, FT, PT, FC, and other traits were investigated in the field. At harvest, 5 plants (excluding marginal effects) were randomly selected from each plot to investigate PH, NPP, TNS, SWP, and other traits. Wanshen SC-G automatic seed testing analyzer was used for seed testing, including for HSW, SL, and SW, which was repeated 3 times.

### 4.4. Determination of Grain Quality Traits

#### 4.4.1. Protein Content

The protein content in seeds was determined by Urumqi Puni Testing Technology Co., Ltd. (Urumqi, China) in accordance with GB5009.5-201 and GB5009.6-2016 standards.

#### 4.4.2. Fatty Acid Content

The fatty acid content of chickpea seeds was determined by the Agricultural Products Quality Supervision and Testing Center of the Ministry of Agriculture (Urumqi) according to the GB/T17377-2008 standard.

#### 4.4.3. Amylum Content, Soluble Solid Concentration Content

The total starch content of chickpea grains was determined by plant starch (Amy) ELISA kit (Jingmei, Jiangsu, China). The soluble solids content was determined according to the national standard GB12295-90 in the laboratory of the Institute of Crop Variety Resources, Xinjiang Academy of Agricultural Sciences.

### 4.5. Statistical and Analytical Processing of Data

The Shannon–Wiener genetic diversity index (*H*’) was used to evaluate the diversity of each trait [30]. According to the mean and standard deviation, the quantitative traits of all materials were divided into 10 grades, that is, grade 1 < x − 2σ to grade 10 ≥ x + 2σ, and the difference between each grade was 0.5σ.

(7) $$H\prime =-\sum P_{i}\times \ln P_{i}$$

*P_i_* is the percentage of the number of materials with grade *i* of a trait in the analysis unit to the total number of materials.

Excel 2021 was used for data processing and analysis, and IBM SPSS Statistics 27.0 was used for principal component analysis and multivariate analysis. We used GraphPad Prism 10.1.2 to create correlation analysis diagrams and violin diagrams. Origin 2021 and R language 4.4.2 were used for correlation, cluster analysis, and corresponding visualizations.

## 5. Conclusions

In this study, a systematic evaluation of 362 chickpea germplasm accessions was conducted based on the main agronomic and quality traits. The germplasm resources exhibited substantial polymorphism in both agronomic and quality characteristics. Multivariate analytical methods, including genetic diversity assessment, correlation analysis, principal component analysis, and cluster analysis, were employed to effectively characterize the genetic structure of the chickpea germplasm. Cluster analysis classified the 362 accessions into five distinct groups. Based on the specific features of each group, 19 accessions with outstanding performance were preliminarily selected. Subsequently, through a multivariate evaluation approach incorporating PCA weighted F-value calculations, 10 elite accessions demonstrating superior performance were identified. These selected accessions can serve as foundational breeding materials for subsequent chickpea variety improvement and cross-breeding programs. This study provides a theoretical basis for the evaluation of chickpea genetic resources and the exploration of functional genes. Although the assessment of agronomic and quality traits offers a simple and rapid means of resource evaluation, these traits are susceptible to environmental influences and human factors, and they offer limited insight into the genetic background of the germplasm. Future research will integrate multi-omics data to elucidate the genetic mechanisms underlying important agronomic traits, with the aim of constructing a breeding system for chickpeas that combines high yield, stress resistance, and superior quality. Such efforts will facilitate genetic improvement and promote the industrial application of chickpea varieties.

## Figures and Tables

**Table 1:** Genetic diversity analysis of 5 qualitative traits of chickpea germplasm resources.

Traits	*H*’	Frequency Distribution (%)							
		1	2	3	4	5	6	7	8
PT	1.123	19.9	17.2	57.4	4.8	0.7	—	—	—
FC	0.760	1.7	76.2	9.7	12.4	—	—	—	—
SS	0.758	72.8	18.3	9.0	—	—	—	—	—
SCC	1.203	3.0	9.0	66.2	9.6	4.5	2.1	0.3	5.4
SSS	0.800	67.4	25.7	6.9	—	—	—	—	—

PT = plant type; FC = flower color; SS = seed shape; SCC = seed coat color; SSS = seed surface shape; “—” indicates that there is no such counting category.

**Table 2:** Genetic diversity analysis of 12 quantitative traits of chickpea germplasm resources.

Traits	Mean	Max	Min	SD	Range	CV (%)	*H*’	*H* ^2^
GP (d)	87.79	105.00	69.00	6.70	36.00	7.63	1.97	53.45
FT (d)	46.37	66.00	30.00	6.51	36.00	14.27	1.45	61.83
PH (cm)	51.24	94.27	23.67	12.82	70.60	25.01	2.07	63.55
PL (mm)	21.61	31.81	12.66	2.35	19.15	10.88	2.05	85.76
PW (mm)	11.12	14.23	6.39	1.38	7.84	12.40	1.61	90.31
NPP	73.89	173.02	10.00	26.51	163.02	35.88	2.00	77.37
NSP	1.19	2.08	0.83	0.26	1.25	22.21	2.05	83.41
TNS	89.08	242.67	10.67	35.11	232.00	39.41	2.00	79.64
SWP (g)	22.62	63.5	2.20	9.43	61.33	41.69	2.01	90.61
HSW (g)	26.23	60.76	8.64	9.31	52.12	35.50	2.04	92.40.
SL (mm)	7.85	11.09	5.68	0.85	5.41	10.88	2.03	90.70
SW (mm)	6.67	9.13	4.59	0.90	4.54	13.51	1.97	80.58

**Table 3:** Multivariate analysis of variance between 12 agronomic traits and years and regions.

Traits	Years		Locations	
	F	*p* Value	F	*p* Value
GP (d)	0.621	−0.454	0.206	0.245
FT (d)	0.236	−0.444	0.646	0.101
PH (cm)	0.708	−0.413	0.228	0.249
PL (mm)	−0.167	0.717	0.608	0.078
PW (mm)	0.070	0.736	0.598	0.086
NPP	−0.747	0.103	−0.255	0.470
NSP	−0.751	0.013	0.215	−0.308
TNS	−0.895	0.100	−0.07	0.305
SWP (g)	−0.07	0.513	−0.385	0.454
HSW (g)	0.875	0.333	−0.243	−0.039
SL (mm)	0.793	0.445	−0.194	−0.092
SW (mm)	0.895	0.344	−0.202	−0.041

The years refer to 2023 and 2024. The location refers to the two regions of Urumqi and Baicheng in Xinjiang, China; F refers to the ratio of the variance between groups to the variance within groups. If *p* ≤ 0.05, the null hypothesis is rejected and the factor is considered to have a significant impact on the dependent variable.

**Table 4:** Principal component analysis of 12 quantitative traits and 4 quality traits.

Traits	Principal Components				
	1	2	3	4	5
GP (d)	0.621	−0.454	0.206	0.245	−0.021
FT (d)	0.236	−0.444	0.646	0.101	−0.080
PH (cm)	0.708	−0.413	0.228	0.249	−0.010
PL (mm)	−0.167	0.717	0.608	0.078	0.160
PW (mm)	0.070	0.736	0.598	0.086	0.157
NPP	−0.747	0.103	−0.255	0.470	−0.127
NSP	−0.751	0.013	0.215	−0.308	0.170
TNS	−0.895	0.100	−0.07	0.305	−0.024
SWP (g)	−0.07	0.513	−0.385	0.454	−0.301
HSW (g)	0.875	0.333	−0.243	−0.039	0.037
SL (mm)	0.793	0.445	−0.194	−0.092	0.004
SW (mm)	0.895	0.344	−0.202	−0.041	−0.012
PC (g/100 g)	0.119	−0.376	0.033	0.621	0.106
FC (g/100 g)	−0.066	−0.058	−0.276	0.123	0.702
AC (g/00 g)	−0.111	0.050	0.190	−0.209	−0.591
SSCC (%)	0.216	0.243	0.208	0.619	−0.046
Eigenvalue	5.177	2.545	1.798	1.599	1.050
Contribution rate (%)	32.358	15.908	11.240	9.993	6.565
Cumulative contribution rate (%)	32.358	48.266	59.506	69.499	76.064

PC = protein content; FC = fat content; AC = Amy content; SSCC = soluble solids concentration content.

**Table 5:** Top 10 comprehensive evaluation results of chickpea germplasm resources.

Number	Variety Name	Origin	Type	Comprehensive Score
YZD133	TaYin YZD-2012-22	Tadzhikistan	kabuli	0.483
YZD353	MuLei (India)	India	kabuli	0.471
YZD109	21412 (B)	ICARDA	desi	0.442
YZD085	Nepal-2	Nepal	kabuli	0.433
YZD077	PinYing 1	Xinjiang, China	kabuli	0.429
YZD083	MuYing 1	Xinjiang, China	kabuli	0.417
YZD359	Y Zi 14-124-132	Yunnan, China	kabuli	0.415
YZD354	Mu Lei Dali	Xinjiang, China	kabuli	0.409
YZD061	21302	National Crop Germplasm Repository	kabuli	0.406
YZD248	L657	National Crop Germplasm Repository	kabuli	0.404

**Table 6:** Investigation standards and assignment of main agronomic traits of chickpea germplasm resources.

Traits	Standards and Assignment of Values for Trait Investigation
GP (d)	The number of days from the second day of sowing to the maturity of the plants
FT (d)	From the second day of sowing to the number of days when the plants flower
PH (cm)	The vertical distance from the leaf node to the top of the main stem of a mature plant
PL (mm)	The length of a mature plant pod from base to tip
PW (mm)	The maximum width of the pod of a mature plant
NPP	During the maturity period, observe and count the number of mature pods contained in each plant
NSP	Observe and count the number of dry grains contained in each pod during the maturation period
TNS	Number of grains per mature plant
SWP (g)	The weight of seeds contained in a single mature plant
HSW (g)	The weight of 100 mature dry seeds
SL (mm)	The length of dry seed from the base to the top of mature plants
SW (mm)	The width of the widest part of the dry seed of a mature plant
PT	1, Upright; 2, semi-upright; 3, semi-spread; 4, spread; 5, prostrate
FC	1, White background with pink veins; 2, white; 3, pink; 4, purplish red
SS	1, Eagle-shaped; 2, sheep-shaped; 3, spherical
SCC	1, Ivory white; 2, light brown; 3, beige; 4, brown; 5, reddish-brown; 6, black; 7, yellow; 8, beige
SSS	1, Rough; 2, uneven; 3, smooth

## Data Availability

The data provided in this study are available upon request from the corresponding author. Due to privacy concerns, these data are not publicly available.

## Supplementary Materials

The following supporting information can be downloaded at https://www.mdpi.com/article/10.3390/plants15010024/s1. Table S1: List of 362 chickpea germplasm resources.

## Abbreviations

The following abbreviations are used in this manuscript:

PCA	Principal component analysis
CV	Coefficient of variation
*H*′	Genetic diversity index
*h*^2^	Narrow-sense heritability
GP	Growth period
FT	Flowering time
PH	Plant height
NPP	Number of pods per plant
NSP	Number of Seeds per Pod
TNS	Total number of seeds per plant
SWP	Seed weight per plant
HSW	Hundred-seed weight
PL	Pod Length
PW	Pod Width
SL	Seed Length
SW	Seed Width
PT	Plant type
FC	Flower color
LC	Leaf Color
SS	Seed shape
SCC	Seed coat color
SSS	Seed surface shape
PC	Protein content
FC	Fat content
AC	Amy content
SSCC	Soluble solid concentration content
*H*^2^	Broad-sense heritability
